# Effects of Homocysteine on white matter diffusion parameters in Alzheimer’s disease

**DOI:** 10.1186/s12883-017-0970-7

**Published:** 2017-10-06

**Authors:** Chen-Chang Lee, Shih-Wei Hsu, Chi-Wei Huang, Wen-Neng Chang, Sz-Fan Chen, Ming-Kung Wu, Chiung-Chih Chang, Lain-Chyr Hwang, Po-Chou Chen

**Affiliations:** 1 Department of Biomedical Engineering, I-Shou University, Kaohsiung, Taiwan; 2 Department of Radiology, Kaohsiung Chang Gung Memorial Hospital, Chang Gung University College of Medicine, Kaohsiung, Taiwan; 3 Department of Neurology, Cognition and Aging Center, Kaohsiung Chang Gung Memorial Hospital, Chang Gung University College of Medicine, Kaohsiung, Taiwan; 4 Department of Psychiatry, Kaohsiung Chang Gung Memorial Hospital, Chang Gung University College of Medicine, Kaohsiung, Taiwan; 5 Department of Electrical Engineering, I-Shou University, Kaohsiung, Taiwan

**Keywords:** Alzheimer’s disease, Homocysteine, Diffusion parameters, Default-mode network

## Abstract

**Background:**

The clinical features of Alzheimer’s disease (AD) are related to brain network degeneration, and hyperhomocysteinemia is related to greater white matter hyperintensities. We investigated the changes in four diffusion tensor imaging parameters in the white matter of patients with early stage AD, examined their associations with homocysteine level, and tested the clinical significance of the diffusion tensor imaging parameters and homocysteine level in correlation analysis with cognitive test scores.

**Methods:**

We enrolled 132 patients with AD and analyzed white matter (WM) macrostructural changes using diffusion tensor neuroimaging parameters including fractional anisotropy (FA), mean diffusion (MD), axial diffusivity (axial-D) and radial diffusivity (RD). Two neuroimaging post-processing analyses were performed to provide complementary data. First, we calculated 11 major bundle microstructural integrities using a WM parcellation algorithm, and correlated them with serum homocysteine levels to explore whether the fiber bundles were affected by homocysteine. Second, we used tract-based spatial statistics to explore the anatomical regions associated with homocysteine levels. Changes in cognitive test scores caused by homocysteine served as the major outcome factor.

**Results:**

The results suggested that homocysteine levels did not have a direct impact on cross-sectional cognitive test scores, but that they were inversely correlated with renal function, B12 and folate levels. Topographies showing independent correlations with homocysteine in FA and MD were more diffusely located compared to the posterior brain regions in axial-D and RD. In the association bundle analysis, homocysteine levels were significantly correlated with the four diffusion parameters even after correcting for confounders, however no association between homocysteine and WM to predict cognitive outcomes was established.

**Conclusions:**

In our patients with AD, homocysteine levels were associated with renal dysfunction and decreased levels of vitamin B12 and folate, all of which require clinical attention as they may have been associated with impaired WM microstructural integrity and modulated cognitive performance in cross-sectional observations.

## Background

The characteristic imaging and pathological findings of Alzheimer’s disease (AD) are cortical atrophy with deposition of plaques and tangles [1]. However, in recent years, cerebral white matter (WM) damage has been reported to be of adverse effect on modulating clinical symptoms in patients with AD [2–4]. Fluid attenuation inversion recovery magnetic resonance imaging (MRI) is widely used to investigate the WM macrostructural damage especially white matter hyperintensities (WMHs). Recent diffusion tensor imaging techniques has shown promise in quantification of WM microstructural changes using fractional anisotropy (FA) and mean diffusivity (MD) [5]. In addition, other diffusion parameters such as axial diffusivity (axial-D) and radial diffusivity (RD) may imply different degree of axonal damage and myelin disruption [6]. Tractography is another diffusion tensor imaging allows tracing neural fibers. The fiber integrity is able to be estimated with the method of a WM parcellation algorithm which allows for the approximation of 3D trajectories of major WM bundles by probabilistic maps [7]. Tract correlation studies can be performed with automated tract-specific quantification of FA (or MD, axial-D, RD). Along with tract-based spatial statistics (TBSS) analysis, the contribution of risk factors to micro-structural changes of the WM in terms of topography and bundle integrity can be quantified and modeled.

The presence of WMHs in AD has been associated with disruption of the blood-brain barrier during aging [8], vasculopathies caused by inflammation [9], impaired cerebral auto-regulation [10], and more severe homocysteinemia [3, 11]. Intensive investigations are underway into the potential mechanisms of biomarker-based neurobiology [3, 9, 11]. Studies on the impact of hyperhomocysteinemia with regards to changes in the WM microstructure and fiber integrity or relationships with cognitive measurements may address whether the target risk biomarkers are of clinical relevance in patients with AD. Based on a literature review [12, 13], we hypothesized that hyperhomocysteinemia in late-onset AD may lead to WM microstructural changes and thus poorer cognitive performance. In this study, we used multi-modal diffusion parameters to identify the primary pathoanatomic correlations of hyperhomocysteinemia with WM microstructural changes, The independent predictive value of hyperhomocysteinemia for the location of WM microstructural changes and tract integrity were analyzed through controlling for known cerebrovascular risk factors.

## Methods

This study was conducted in accordance with the Declaration of Helsinki. It was approved by the Institutional Review Board of Chang Gung Memorial Hospital. The participants were followed up at the Cognition and Aging Center, Department of General Neurology, Kaohsiung Chang Gung Memorial Hospital. After the consensus of a panel composed of neuroradiologists, neurologists and neuropsychologists, a total of 132 subjects (69 females, 63 males) were enrolled [9]. These subjects were diagnosed with AD according to the International Working Group criteria [14] with a clinical diagnosis of typical AD. The patients all had a clinical dementia rating score of 0.5 or 1. They were under acetylcholine esterase inhibitor treatment in a stable condition. The exclusion criteria were a past history of clinical stroke and depression and a modified Hachinski ischemic score > 4.

### Clinical and neurobehavioral assessments

The demographic data of each patient were recorded after enrollment. The neurobehavioral tests were administered by a trained neuro-psychologist. A global assessment of cognitive function was performed using Mini-Mental State Examination (MMSE) scores [15] and Cognitive Ability-Screening Instrument (CASI) [16]. The combination of mental manipulation, attention, abstract thinking and verbal fluency sub-domain scores of the CASI were used to assess executive function (CASI EFT) [11], while other cognitive domains included orientation, short term memory, long term memory, language and drawing.

### Homocysteine and Apolipoprotein E genotype analysis

Antecubital venous blood samples were collected in evacuated tubes containing EDTA after overnight fasting for 8 h and centrifuged for 10 min before plasma biomarker concentrations were measured. Plasma Homocysteine was measured using an IMx florescence polarization immuno-assay analyzer (Abbott Laboratories, Chicago, IL), with intra- and inter-assay coefficients of variation of 3% and 5%, respectively. The minimum detection level was 0.7 umol/L. The Apolipoprotein E genotype was determined by polymerase chain reaction-restriction fragment length polymorphism assay and restriction enzyme HhaI [17]. Those with the presence of one or two Apolipoprotein E 4 alleles were defined as Apolipoprotein E 4 carriers.

### Cerebrovascular risk confounders

Neuroinflammation, metabolic disorders and oxidative stress have been reported to be associated with more severe WMHs in AD [9]. Therefore, in addition to homocysteine, the following risk confounders were also included as cerebrovascular risk confounders in the analysis: age, hemoglobin, high sensitive C-reactive protein, total cholesterol, triglycerol, high-density lipoprotein, low-density lipoprotein, creatinine, vitamin B12, folate, and hemoglobin-A1C [18]. The estimated glomerular filtration rate (eGFR) was calculated using the abbreviated Modification of Diet in Renal Disease [19].

### Image acquisition

MR images were acquired using a 3.0 T MRI scanner (Excite, GE Medical Systems, Milwaukee, WI, USA), and diffusion-tensor imaging was acquired using the following parameters: repetition time = 9600 ms, echo time = 62.7 ms, flip angle = 90°, a 192 × 192 mm field of view, a 128 × 128 matrix and a 4-mm axial slice thickness. A total 40 contiguous axial slices were obtained to cover the whole brain. The b value was 1000 s/mm^2^. Diffusion-weighted gradients were applied in 61 non-collinear directions, optimized using a static electron-repulsion model. One reference image was acquired using the same imaging parameters but without diffusion weighting.

In this study we used TBSS and tractography to evaluate WM microstructural changes. TBSS is a voxelwise and skeleton-based method to detect anatomical changes between groups or correlations with biomarkers. For tractography, we treated each fiber bundle as an independent parameter to evaluate their association with hyperhomocysteinemia or cognitive function. Specific to this research protocol, the TBSS results were taken to reflect regional changes as opposed to the tractography results which were taken to reflect structural connection alterations.

### TBSS for topographies of Homocysteine levels

To investigate the impact of homocysteine we used TBSS for topography analysis (FSL software version 5.0.1; www.fmrib.ox.ac.uk/fsl/). In brief, the FA image of each subject was aligned to a target FA image and transformed into the common space by affine registration. Mean FA skeleton were created from the images of all the participants. The FA images from all participants were subsequently projected onto a mean FA skeleton. For voxel-wise statistical analysis, 4D projected FA images were put into a general linear model to find voxels that were correlated with the covariates of interest adjusted for age. The projection vectors from each individual participant were estimated onto the mean FA skeleton and then the data for MD, axial-D and RD images were generated by applying non-linear warps and skeleton projections. The resulting statistical maps were analyzed at a threshold of *p* < 0.05 using the threshold-free cluster enhancement method for multiple comparisons (http://fsl.fmrib.ox.ac.uk/fsl/fslwiki/randomise).

### WM tract parcellation for 11 associated bundles

As the clinical symptomatology depends on the structural integrity, we also tested the impact of homocysteine on major fiber tracts. A WM parcellation algorithm was used to calculate 11 major bundles following the procedure reported by Hua et al. [7]. The value of FA, MD, axial-D and RD in these 11major bundles was used to assess the microstructural integrities of the WM bundles. The tracts being affected by homocysteine were also correlated with the fiber tract integrities or cognitive test scores, with or without adjustments for confounders, to understand the clinical significance.

### Statistical analysis

Demographic and laboratory data were expressed as mean ± standard deviation, and Pearson’s correlation was used to test the correlations between continuous variables. Partial correlation analysis was used to adjust for possible confounders that showed co-linearity with homocysteine. FSL was used for voxel-wise analysis, while the statistical analyses of the clinical and laboratory data were conducted using SPSS software (SPSS version 22 for Windows®, SPSS Inc., Chicago, IL). To balance type I and type II errors after multiple comparisons, a two-tailed *p* value of less than 0.01 was considered to be statistically significant.

## Results

### Clinical data and biomarkers levels

Table 1 shows the demographic and biomarker data of the patients with AD and the correlations between homocysteine and serum biomarkers. There were significant relationships between homocysteine and creatinine, eGFR, folate and vitamin B12 levels. Of note, although 118 patients had creatinine levels within the reference range of <1.2 mg%, only 22 patients fulfilled the criteria for a normal eGFR (> 90 ml/min). homocysteine levels were not correlated to any of the selected cognitive test scores. Un addition, there was no significant difference in homocysteine level between the E4 carriers (*n* = 58; 12.2 ± 4.1) and non-E4 carriers (*n* = 74; 13.0 ± 4.6, *p* = 0.29).

**Table 1:** Demographical characteristics and neuropsychiatric tests in 132 Alzheimer’s disease

	Mean	±	SD	Hcy correlation※	*P* value
Age	73.5	±	7.5	0.12	0.19
Education (year)	7.2	±	4.8	0.07	0.44
Apolipoprotein E4 carrier (positive case, %)	58 (43.9%)			–	
Sex (male/female)	63/69			–	
Mini-Mental State Examination	20.1	±	6.5	0.00294	0.97
CASI total scores	67.4	±	21.1	0.01	0.89
CASI executive function test scores	25.1	±	8.3	−0.01	0.87
CASI Subdomains		±			
Short Term Memory	5.4	±	3.7	0.00087	0.99
Orientation	12.7	±	5.3	−0.02	0.86
Long Term Memory	8.3	±	2.5	0.08	0.36
Language	8.1	±	2.3	0.01	0.93
Drawing	7.9	±	2.8	0.10	0.26
Attention	6.3	±	1.4	−0.04	0.65
Verbal fluency	5.2	±	2.8	−0.06	0.51
Abstract thinking	8.1	±	2.9	0.02	0.85
Mental manipulation	5.5	±	3.3	0.01	0.88
Cerebrovascular Risk Biomarkers					
High sensitive C reactive protein (mg/L)	2.7	±	4.9	−0.13	0.14
Homocysteine [Hcy] (umol/L)	12.7	±	4.4	**–**	**–**
Hemoglobin-A1C (**%)**	6.2	±	1.1	0.00069	0.99
Creatinine (mg%)	0.9	±	0.4	0.54	3.7 × 10^−11^
Glomerular filtration rate (mL/min)	74.7	±	20.5	−0.47	1.4 × 10^−8^
high-density lipoprotein (mg/dl)	58.0	±	17.0	−0.22	0.01
low-density lipoprotein (mg/dl)	104.8	±	36.6	0.01	0.95
Hemoglobin (mg/dl)	13.4	±	1.6	0.01	0.93
Total Cholesterol (mg/dl)	187.5	±	39.9	−0.07	0.43
Triglyceride (mg/dl)	119.3	±	60.6	0.09	0.31
Vitamin B12 (pg/dl)	608.4	±	341.1	−0.36	2.7 × 10^−5^
Folate (ng/dl)	12.2	±	5.6	−0.40	1.6 × 10^−6^

Data are presented as mean (standard deviation) or number (percentage; %)

*Abbreviations*: *CASI* Cognitive Ability Screening Instrument

Attention, verbal fluency, abstract thinking, and mental manipulation sub-domain scores of the CASI were added to assess executive function; APOE4 carriers were defined as the presence of one or two APOE4 alleles. The Glomerular filtration rate is calculated by the Modification of Diet in Renal Diseaseformula; ※Pearson correlation coefficient

### Topography and fiber integrities using diffusion parameters to assess the impact of homocysteine

**Fig. 1:**
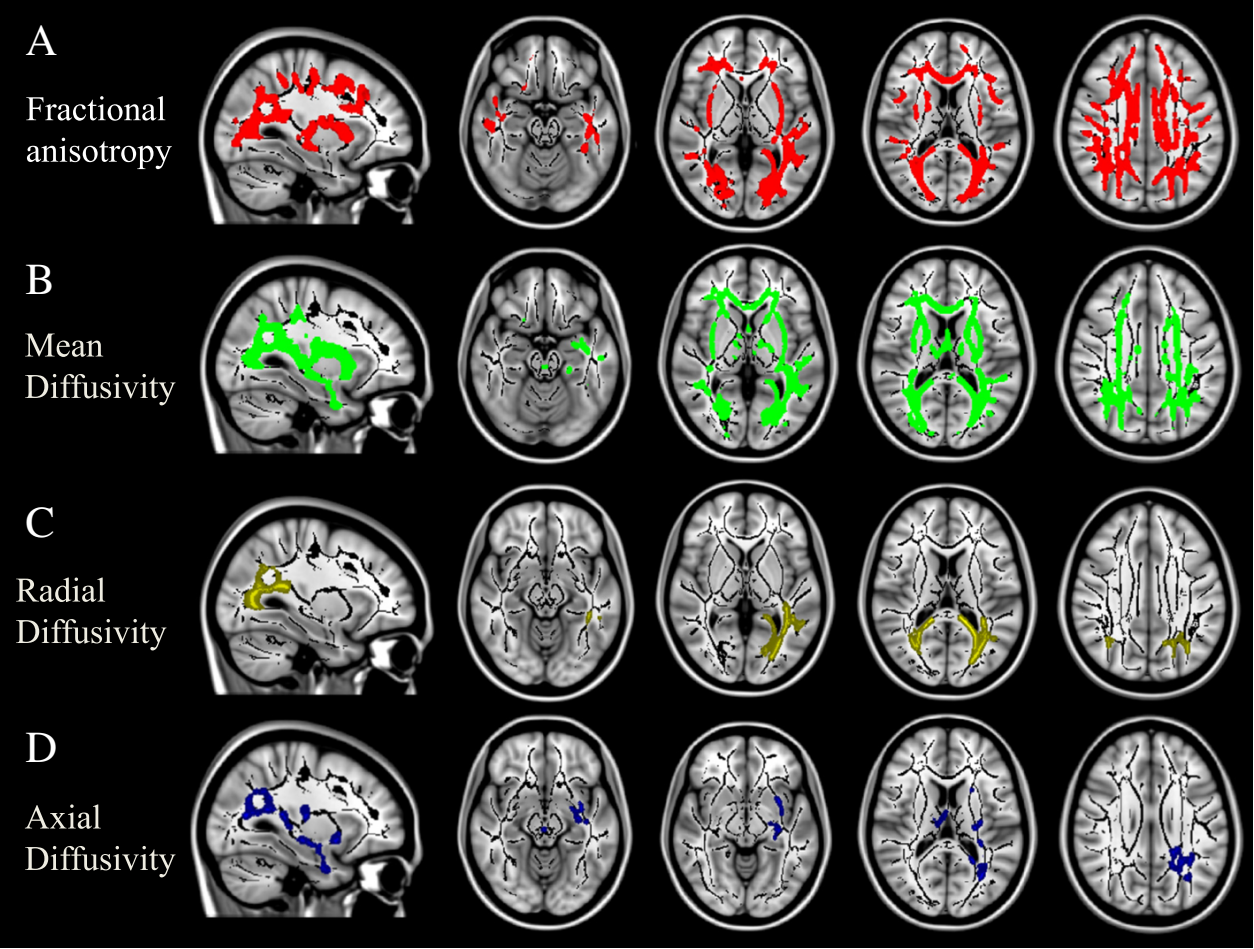
White matter topographies related to serum homocysteine levels. **a** Fractional anisotropy: red; (**b**) Mean diffusivity: green; (**c**) Radial diffusivity; (**d**) Axial diffusivity. The images are displayed on a standard brain render with the threshold of *p* < 0.05 (threshold-free cluster enhancement method for multiple comparisons). The underlying black represents the white matter skeleton. Adjustments were made for serum creatinine and glomerular filtration rate according to the Modification of Diet in Renal Disease formula, vitamin B12 and folate levels

The anatomical topographies of homocysteine adjusted for confounders are shown in Fig. 1. The involvements of FA (Fig. 1a, red) and MD (Fig. 1b, green) were more generalized compared to RD (Fig. 1c, brown) and axial-D (Fig. 1d, blue), which were more posteriorly oriented.

We also explored whether homocysteine levels were directly related to WM integrity using four different diffusion parameters (Table 2). The results showed that homocysteine was associated with nearly all the WM integrities except for the hippocampal cingulum bundle and the axial-D in the forceps minor and uncinate fasciculus bundles.

**Table 2:** Comparisons of diffusion parameters using 11 association Fibers

Fiber name	Mean ± SD			Hcy	Hcy Adjusted^a^
Forceps.major					
Fractional Anisotropy	0.475	±	0.041	−0.415*	−0.358*
Axial Diffusivity(×10^−3^)	1.487	±	0.136	0.227*	0.060
Radial Diffusivity(×10^−3^)	0.696	±	0.129	0.339*	0.214
Mean Diffusivity(×10^−3^)	0.960	±	0.128	0.307*	0.164
Forceps.minor					
Fractional Anisotropy	0.351	±	0.026	−0.351*	−0.258*
Axial Diffusivity(×10^−3^)	1.415	±	0.066	0.216	0.053
Radial Diffusivity(×10^−3^)	0.820	±	0.070	0.328*	0.173
Mean Diffusivity(×10^−3^)	1.018	±	0.067	0.301*	0.139
Anterior.thalamic.radiation					
Fractional Anisotropy	0.605	±	0.056	−0.339*	−0.248*
Axial Diffusivity(×10^−3^)	3.052	±	0.440	0.346*	0.179
Radial Diffusivity(×10^−3^)	2.049	±	0.414	0.356*	0.198
Mean Diffusivity(×10^−3^)	2.383	±	0.422	0.353*	0.192
Corticospinal.tract					
Fractional Anisotropy	0.992	±	0.055	−0.282*	−0.302*
Axial Diffusivity(×10^−3^)	2.619	±	0.111	0.456*	0.349*
Radial Diffusivity(×10^−3^)	1.191	±	0.118	0.442*	0.388*
Mean Diffusivity(×10^−3^)	1.667	±	0.110	0.467*	0.393*
Cingulum._cingulate.gyrus					
Fractional Anisotropy	0.677	±	0.062	−0.329*	−0.231*
Axial Diffusivity(×10^−3^)	2.272	±	0.091	−0.053	−0.022
Radial Diffusivity(×10^−3^)	1.379	±	0.105	0.214*	0.154
Mean Diffusivity(×10^−3^)	1.677	±	0.093	0.143*	0.108
Cingulum._hippocampus					
Fractional Anisotropy	0.578	±	0.068	−0.177	−0.102
Axial Diffusivity(×10^−3^)	2.347	±	0.245	0.162	0.039*
Radial Diffusivity(×10^−3^)	1.540	±	0.231	0.177	0.066
Mean Diffusivity(×10^−3^)	1.809	±	0.233	0.173	0.057
Inferior.frontooccipital.fasciculus					
Fractional Anisotropy	0.736	±	0.053	−0.364*	−0.343*
Axial Diffusivity(×10^−3^)	2.554	±	0.133	0.392*	0.285*
Radial Diffusivity(×10^−3^)	1.432	±	0.148	0.432*	0.357*
Mean Diffusivity(×10^−3^)	1.806	±	0.141	0.427*	0.340*
Inferior.longitudinal.fasciculus					
Fractional Anisotropy	0.744	±	0.050	−0.343*	−0.292*
Axial Diffusivity(×10^−3^)	2.413	±	0.125	0.243*	0.186
Radial Diffusivity(×10^−3^)	1.350	±	0.126	0.343*	0.272*
Mean Diffusivity(×10^−3^)	1.704	±	0.123	0.317*	0.249*
Superior.longitudinal.fasciculus					
Fractional Anisotropy	0.631	±	0.049	−0.266*	−0.281*
Axial Diffusivity(×10^−3^)	2.274	±	0.136	0.343*	0.218
Radial Diffusivity(×10^−3^)	1.456	±	0.140	0.356*	0.275*
Mean Diffusivity(×10^−3^)	1.729	±	0.137	0.357*	0.261*
Uncinate.fasciculus					
Fractional Anisotropy	0.680	±	0.050	−0.282*	−0.246*
Axial Diffusivity(×10^−3^)	2.664	±	0.265	0.218	0.092
Radial Diffusivity(×10^−3^)	1.625	±	0.250	0.254*	0.152
Mean Diffusivity(×10^−3^)	1.971	±	0.253	0.243*	0.132
Superior.longitudinal.fasciculus temporal.part					
Fractional Anisotropy	0.898	±	0.084	−0.321*	−0.398*
Axial Diffusivity(×10^−3^)	2.473	±	0.147	0.344*	0.293*
Radial Diffusivity(×10^−3^)	1.179	±	0.146	0.445*	0.466*
Mean Diffusivity(×10^−3^)	1.610	±	0.136	0.441*	0.438*

*p< 0.01

^a^adjusted for glomerular filtration rate, creatinine, folate and vitamine B12 levels

We then adjusted for the factors that may have confounded the homocysteine levels including eGFR, creatinine, folate and vitamin B12 levels (Table 2). The rationale for adding these confounders was to understand whether the impact of homocysteine on the diffusion parameters was independent or modulated by these factors. Some of the fibers that initially showed significance disappeared, including FA of the hippocampal cingulum bundle, axial-D of the forceps major and minor, and uncinate fasciculus, RD of the forceps minor, cingulum bundles of the anterior cingulate, hippocampus, and uncinate fasciculus, and MD of the forceps major, forceps minor, cingulum bundle of the anterior cingulate, and uncinate fasciculus.

### WM integrities affected by homocysteine and related confounders for cognitive outcomes

In AD, the WM integrity may determine the cognitive outcome measures. To understand whether the impact of WM integrity on cognitive function was through homocysteine and its related biomarkers, we further adjusted for them in the correlation analysis between WM integrities and cognitive measures.

For FA, most of the selected WM bundles were significantly correlated with MMSE, CASI total scores, CASI EFT and short-term memory scores (Table 3) before and after the adjustments. In simple correlation analysis, the relationship between the temporal part of the superior longitudinal fasciculus and MMSE was not significant. However, after adjusting for homocysteine and related confounders, significance was found.

**Table 3:** Cognitive outcomes in relation to white matter integrities using fractional anisotropy adjusted for confounding factors

Tract name	Simple correlation				Adjusted for confounders^#^			
	MMSE	CASI total	CASI EFT	STM	MMSE	CASI total	CASI EFT	STM
Forceps.major	.284(*)	.294(*)	.358(*)	.246(*)	.329(*)	.342(*)	.396(*)	.278(*)
Forceps.minor	.291(*)	.300(*)	.364(*)	.252(*)	.333(*)	.342(*)	.395(*)	.285(*)
Anterior.thalamic.radiation	.271(*)	.276(*)	.333(*)	.241(*)	.298(*)	.302(*)	.350(*)	.266(*)
Corticospinal.tract	.304(*)	.308(*)	.361(*)	.249(*)	.316(*)	.331(*)	.379(*)	.252(*)
Cingulum._cingulate.gyrus	.308(*)	.322(*)	.342(*)	.280(*)	.327(*)	.344(*)	.355(*)	.289(*)
Cingulum._hippocampus	.351(*)	.384(*)	.344(*)	.451(*)	.342(*)	.375(*)	.334(*)	.447(*)
Inferior.frontooccipital.fasciculus	.222	.241(*)	.314(*)	.213	.251(*)	.280(*)	.343(*)	.230(*)
Inferior.longitudinal.fasciculus	.266(*)	.290(*)	.350(*)	.214	.216	.240(*)	.280(*)	.193
Superior.longitudinal.fasciculus	.199	.219	.269(*)	.182	.252(*)	.260(*)	.263(*)	.252(*)
Uncinate.fasciculus	.241(*)	.243(*)	.256(*)	.251(*)	.295(*)	.323(*)	.372(*)	.230(*)
Superior.longitudinal.fasciculus.temporal.part	.161	.194	.248(*)	.103	.198	.241(*)	.279(*)	.128

Numbers indicate Correlation Coefficient;

*Correlation is significant at the 0.01 level (2-tailed)

^#^Adjusted for homocysteine, vitamine B12, folate, creatinine, glomerular filtration rate;

*MMSE* minimental state examination, *CASI* Cognitive Ability Screening Instrument, *EFT* Attention, verbal fluency, abstract thinking, and mental manipulation sub-domain scores of the CASI were added to assess executive function, *STM* short term memory from CASI

The data for axial-D and cognitive tests are shown in Table 4. Similar to the data for FA, most of the selected WM bundles were significantly correlated with MMSE, CASI total scores, CASI EFT and short-term memory scores before and after the adjustments.

**Table 4:** Cognitive outcomes in relation to white matter integrities using axial diffusivities adjusted for confounding factors

Tract name	Simple correlation				Adjusted for confounders^#^			
	MMSE	CASI total	CASI EFT	STM	MMSE	CASI total	CASI EFT	STM
Forceps.major	−.220	−.213	−.207	−.138	−.228(*)	−.210	−.201	−.139
Forceps.minor	−.324(*)	−.325(*)	−.341(*)	−.250(*)	−.322(*)	−.320(*)	−.339(*)	−.242(*)
Anterior.thalamic.radiation	−.297(*)	−.306(*)	−.337(*)	−.273(*)	−.331(*)	−.335(*)	−.359(*)	−.308(*)
Corticospinal.tract	−.154	−.132	−.189	−.034	−.183	−.159	−.207	−.055
Cingulum._cingulate.gyrus	−.374(*)	−.387(*)	−.365(*)	−.314(*)	−.353(*)	−.368(*)	−.355(*)	−.294(*)
Cingulum._hippocampus	−.463(*)	−.468(*)	−.385(*)	−.486(*)	−.467(*)	−.468(*)	−.380(*)	−.484(*)
Inferior.frontooccipital.fasciculus	−.372(*)	−.350(*)	−.379(*)	−.241(*)	−.398(*)	−.379(*)	−.400(*)	−.250(*)
Inferior.longitudinal.fasciculus	−.430(*)	−.409(*)	−.371(*)	−.323(*)	−.443(*)	−.425(*)	−.377(*)	−.318(*)
Superior.longitudinal.fasciculus	−.392(*)	−.375(*)	−.363(*)	−.286(*)	−.416(*)	−.397(*)	−.376(*)	−.293(*)
Uncinate.fasciculus	−.319(*)	−.335(*)	−.294(*)	−.352(*)	−.331(*)	−.345(*)	−.296(*)	−.357(*)
Superior.longitudinal.fasciculus.temporal.part	−.234(*)	−.216	−.210	−.129	−.246(*)	−.234(*)	−.217	−.134

Numbers indicate Correlation Coefficient;

*Correlation is significant at the 0.01 level (2-tailed)

^#^Adjusted for homocysteine, vitamine B12, folate, creatinine, glomerular filtration rate

*MMSE* minimental state examination, *CASI* Cognitive Ability Screening Instrument, *EFT* Attention, verbal fluency, abstract thinking, and mental manipulation sub-domain scores of the CASI were added to assess executive function, *STM* short term memory from CASI

The data for RD and cognitive tests are shown in Table 5. Nearly all the selected WM bundles were significantly correlated with MMSE, CASI total scores, CASI EFT and short-term memory scores before and after the adjustments. The inverse correlation coefficients showed an increasing trend after adjustments for the confounders.

**Table 5:** Cognitive outcomes in relation to white matter integrities using radial diffusivities adjusted for confounding factors

Tract name	Simple correlation				Adjusted for confounders^#^			
	MMSE	CASI total	CASI EFT	STM	MMSE	CASI total	CASI EFT	STM
Forceps.major	−.269(*)	−.271(*)	−.296(*)	−.205	−.295(*)	−.290(*)	−.308(*)	−.220
Forceps.minor	−.357(*)	−.365(*)	−.405(*)	−.300(*)	−.383(*)	−.389(*)	−.423(*)	−.318(*)
Anterior.thalamic.radiation	−.302(*)	−.313(*)	−.349(*)	−.276(*)	−.340(*)	−.345(*)	−.373(*)	−.315(*)
Corticospinal.tract	−.273(*)	−.268(*)	−.325(*)	−.178	−.309(*)	−.310(*)	−.358(*)	−.203
Cingulum._cingulate.gyrus	−.430(*)	−.450(*)	−.459(*)	−.362(*)	−.430(*)	−.452(*)	−.458(*)	−.356(*)
Cingulum._hippocampus	−.471(*)	−.488(*)	−.419(*)	−.519(*)	−.474(*)	−.488(*)	−.415(*)	−.519(*)
Inferior.frontooccipital.fasciculus	−.321(*)	−.322(*)	−.377(*)	−.244(*)	−.361(*)	−.367(*)	−.413(*)	−.266(*)
Inferior.longitudinal.fasciculus	−.406(*)	−.407(*)	−.414(*)	−.314(*)	−.440(*)	−.444(*)	−.438(*)	−.327(*)
Superior.longitudinal.fasciculus	−.354(*)	−.356(*)	−.367(*)	−.276(*)	−.384(*)	−.386(*)	−.386(*)	−.292(*)
Uncinate.fasciculus	−.321(*)	−.340(*)	−.307(*)	−.360(*)	−.336(*)	−.355(*)	−.313(*)	−.369(*)
Superior.longitudinal.fasciculus.temporal.part	−.232(*)	−.246(*)	−.283(*)	−.139	−.277(*)	−.303(*)	−.324(*)	−.166

Numbers indicate Correlation Coefficient

*Correlation is significant at the 0.01 level (2-tailed)

^#^Adjusted for homocysteine, vitamine B12, folate, creatinine, glomerular filtration rate

*MMSE* minimental state examination, *CASI* Cognitive Ability Screening Instrument, *EFT* Attention, verbal fluency, abstract thinking, and mental manipulation sub-domain scores of the CASI were added to assess executive function, *STM* short term memory from CASI

For MD (Table 6), the significant correlations between short-term memory and the temporal part of the superior longitudinal fasciculus disappeared after adjusting for the confounders. However, the uncinate fasciculus MD was significantly correlated with short-term memory after adjusting for the confounders.

**Table 6:** Cognitive outcomes in relation to white matter integrities using mean diffusivities adjusted for confounding factors

Tract name	Simple correlation				Adjusted for confounders^#^			
	MMSE	CASI total	CASI EFT	STM	MMSE	CASI total	CASI EFT	STM
Forceps.major	−.258(*)	−.257(*)	−.272(*)	−.186	−.278(*)	−.268(*)	−.277(*)	−.195
Forceps.minor	−.356(*)	−.362(*)	−.395(*)	−.292(*)	−.373(*)	−.377(*)	−.406(*)	−.301(*)
Anterior.thalamic.radiation	−.301(*)	−.311(*)	−.346(*)	−.275(*)	−.338(*)	−.342(*)	−.369(*)	−.313(*)
Corticospinal.tract	−.246(*)	−.235(*)	−.294(*)	−.139	−.284(*)	−.276(*)	−.326(*)	−.164
Cingulum._cingulate.gyrus	−.442(*)	−.461(*)	−.461(*)	−.372(*)	−.433(*)	−.454(*)	−.454(*)	−.359(*)
Cingulum._hippocampus	−.474(*)	−.487(*)	−.412(*)	−.514(*)	−.478(*)	−.488(*)	−.409(*)	−.514(*)
Inferior.frontooccipital.fasciculus	−.342(*)	−.336(*)	−.383(*)	−.247(*)	−.379(*)	−.378(*)	−.416(*)	−.265(*)
Inferior.longitudinal.fasciculus	−.424(*)	−.417(*)	−.409(*)	−.324(*)	−.452(*)	−.448(*)	−.428(*)	−.332(*)
Superior.longitudinal.fasciculus	−.372(*)	−.368(*)	−.372(*)	−.284(*)	−.401(*)	−.396(*)	−.389(*)	−.297(*)
Uncinate.fasciculus	−.249(*)	−.253(*)	−.277(*)	−.145	−.337(*)	−.354(*)	−.309(*)	−.367(*)
Superior.longitudinal.fasciculus.temporal.part	−.258(*)	−.257(*)	−.272(*)	−.186	−.288(*)	−.302(*)	−.310(*)	−.167

*Correelation is significant at the 0.01 level (2-tailed)

^#^Adjusted for homocysteine, vitamine B12, folate, creatinine, glomerular filtration rate

*MMSE* minimental state examination, *CASI* Cognitive Ability Screening Instrument, *EFT* Attention, verbal fluency, abstract thinking, and mental manipulation sub-domain scores of the CASI were added to assess executive function, *STM* short term memory from CASI

## Discussion

### Major findings

This study explored the possible roles of homocysteine and related confounders in modulating WM fiber tract integrities and predicting neurobehavioral test scores in patients with AD. There were three major findings. First, there were no correlations between homocysteine levels and cognitive measurements in cross-sectional observations. However, associations between homocysteine level and renal function (creatinine and eGFR), vitamin B12 and folate were found. Second, although the impacts of homocysteine and WM integrities were established, the weighting of the impact of homocysteine (or the related confounders) on the WM may be considered to be clinically minor, as most of the WM tracts predicted the clinical test scores after adjusting for these serum biomarkers. Finally, our results suggest different WM topographies of the four diffusion parameters affected by homocysteine level. The predominant involvement of the posterior part of the brain across all four diffusion parameters, especially increase in axial-D and RD may reflect its pathological nature in AD.

### Homocysteine and WM integrity

Hyperhomocysteinemia has been reported to be a risk factor for cerebral small vessel disease [20] and for developing AD [21]. We explored the topographies that were independently affected by serum levels of homocysteine. Of note, FA and MD, which are two parameters that reflect the general integrity of WM bundles, were diffusely located, in contrast to axial-D and RD that showed a posterior predilection. Our analysis was not able to provide a mechanism to explain why the location of axial-D and RD showed a posterior predilection. However, this relationship may highlight the vulnerability of patients with AD to neuronal degeneration and amyloid pathogenesis [22, 23], and may also strengthen the link between hyperhomocysteinemia and cerebral hypoperfusion [11] and the higher number of WMHs [3] observed in AD.

### Association between WM bundle involvement and hyperhomocysteinemia

Recent neuroscience studies have supported the relation between cognitive function and the architecture of the neural network [24]. As the potential mechanisms of biomarker-genetic-based neurobiology are still under investigation [25–28], studies on the expression of homocysteine may address how variations in serum level may affect the organization of WM integrities. The hypothesis that hyperhomocysteinemia may result in greater WMHs and cognitive decline in AD has been reported based on cerebrovascular events [29] and silent cerebrovascular events with cognitive decline [30]. In the study by Hogervorst et al., the distribution pattern of leukoaraiosis in brain CT was evident, and the cross-sectional MMSE scores were significantly lower in the AD group with moderate to severe leukoaraiosis compared to the group with none to minimal leukoaraiosis [31]. Hyper-homocysteinemia was also an independent risk factor for developing leukoaraiosis in patients with AD even after controlling for known cerebrovascular risk factors [31].

### Homocysteine level was not directly related to cognitive impairment

We did not find associations between homocysteine level and cognitive measurements or an effect of homocysteine on WM that could predict cognitive measurements in our patients with AD. Therefore, although the impact of homocysteine on WM tract integrity was significant, its clinical relevance on cognitive outcomes may be considered as minor. The non-synonymous polymorphism in the methylenetetrahydrofolate reductase gene at position 677 results in reduced enzyme activity, while the common 677 C to T transition in the gene represents a well-identified genetic determinant for hyperhomocysteinemia. Although higher levels of homocysteine have been reported in the T variants [32], several studies have reported that the T variants may not present with worse cognitive test outcomes [8, 33–35]. In addition, one study suggested that the TT genotype may demonstrate decreased processing speed and executive function [36] while another reported opposite findings in that the 677 TT group showed better sensorimotor speed [37]. Moreover, Gussekloo et al. [38] and others [8, 33–35] reported that C677T was not a genetic risk factor for cognitive impairment. Elderly Chinese males with the CT genotype, but without dementia, have been shown to have higher CASI scores than those with the other two homozygotes especially in short term memory and mental manipulation subdomains [39]. Taken together, the association between a higher homocysteine level in T variants and cognitive outcomes is controversial, suggesting that other factors may participate in the outcome measure of modulation.

### Factors that may modulate the impact of homocysteine

The inverse correlation between homocysteine level and B12 and folate has been well established [40] and is consistent with our findings. Hyperhomocysteinemia is commonly found in patients with renal dysfunction [41], and this is particularly relevant in this study as renal dysfunction can be underestimated when evaluated using serum creatinine level in patients with AD. In our patient group with a normal creatinine level, there was a correlation between homocysteine and creatinine level, however most of the patients with AD with a creatinine level within the reference range also had impaired renal function. Many studies [31, 42] have discussed the relationship between hyperhomocysteinemia and WMHs, however few have discussed the confounding effects of renal function, vitamin B12, and folate. Hisayama reported that an elevated serum homocysteine level in the general population was also a significant risk factor for the development of chronic kidney disease [43]. Further longitudinal studies on the complex interactions between biomarkers are required to elucidate the causal relationships and possible treatment strategies. Our correlation analysis showed that WM microintegrity using diffusion parameters was also significantly correlated with homocysteine level, even after considering these confounders.

### Study limitations

There are three limitations to this study. First, the causality of association was based on a cross-sectional design, as all the serum biomarkers were measured at baseline. Therefore, the interpretation of the effect of homocysteine on cognitive test scores cannot be applied to a single patient with longitudinal follow-up. Further studies using a longitudinal design are needed to confirm our observations and the pathogenic role of homocysteine in the development of microstructural WM changes and cognitive impairment in patients with AD. Second, a previous longitudinal study reported that a plasma homocysteine level greater than 14 μmol/L was associated with a nearly two-fold increased risk of AD, although most of their patients had a mean level of homocysteine of 12.7 ± 4.4 μM [44]. We found that homocysteine level may modulate the WM integrity that determines the cognitive outcomes. However, we did not establish a direct relationship between homocysteine and cognitive outcomes. Further studies are needed to investigate whether a wider range of homocysteine levels would have helped to establish a primary relationship between homocysteine and cognitive outcomes. Third, although we considered possible confounders and adjusted for them, biomarkers other than those discussed may also have confounded the study results. For example, endothelial dysfunction has been shown to augment the adverse effects of homocysteine [9]. Fourth, the relationship between homocysteine lowering treatment using vitamin B12 was not fully tested here. Randomized, controlled and longitudinal follow-up studies are warranted to clarify whether such homocysteine lowering treatment can improve cognitive outcomes.

## Conclusion

In our patients with AD, homocysteine levels did not have a main effect on cognitive function. Homocysteine levels were correlated with eGFR, creatinine, folate and vitamin B12 levels, and these factors may modulate the WM microstructural integrity that can predict cognitive performance in cross-sectional observations.

## Abbreviations

AD: Alzheimer’s disease
axial-D: Axial diffusivity
CASI: Cognitive Ability-Screening Instrument
EFT: Executive function
eGFR: Estimated glomerular filtration rate
FA: Fractional anisotropy
MD: Mean diffusion
MMSE: Mini-Mental State Examination
MRI: Magnetic resonance imaging
RD: Radial diffusivity
TBSS: Tract-based spatial statistics
WM: White matter
WMHs: white matter hyperintensities
